# Risk factors associated with age at diagnosis of diabetes among noninstitutionalized US population: the 2015–2016 National Health and Nutrition Examination Survey

**DOI:** 10.1186/s12889-020-09231-1

**Published:** 2020-07-16

**Authors:** Daudet Ilunga Tshiswaka, Chris B. Agala, A. J. Guillory, Breanna Walters, Justice Mbizo

**Affiliations:** 1 Department of Public Health, Usha Kundu, MD College of Health, University of West Florida, 11000 University Parkway, Pensacola, FL 32514 USA; 2 School of Medicine, University of North Carolina at Chapel-Hill, 211B Cameron Ave, Chapel-Hill, NC 27516 USA

**Keywords:** Diabetes, Risk factors, Anthropometric measurements, Physical examination

## Abstract

**Background:**

Demographic and anthropometric factors associated with the age at diagnosis of diabetes have not been extensively studied. Much of the literature using anthropometric measures has been associated with other health factors such as obesity and coronary heart disease. The purpose of this study was to assess the relationship between different sets of anthropometric factors and age of diabetes diagnosis in the United States.

**Methods:**

Using the NHANES 2015–2016 data set, weighted linear regression analysis was performed on observations from 600 qualified individuals with diabetes to study associations between anthropometric and demographic factors and the age of diabetes diagnosis.

**Results:**

Results of our analysis support the evidence of significant relationships between the anthropometric characteristics and demographic factors and age at diabetes diagnosis. Specifically, age at diagnosis of diabetes is predicted to decrease by 1.03 (*p* < 0.01) and 0.91 (*p* < 0.01) years when BMI and upper leg length go up by one unit each, respectively. Similarly, age at diagnosis of diabetes decreases by 0.02 years and by 1.72 years when refrigerated glucose serum increases by 1 mg/dL (*p <* 0.05) and when household size increases by one person, respectively. Male respondents were diagnosed with diabetes 3.41 years later than their female counterparts. Conversely, age at diagnosis of diabetes increases by 1.24 years when the average sagittal abdominal diameter goes up by 1 cm (*p <* 0.05). In addition, Mexican American respondents were diagnosed 5.00 years younger than the non-Hispanic White counterparts.

**Conclusions:**

Our findings show that anthropometric factors, including BMI, refrigerated glucose serum and upper leg length increase have an inverse linear association with age of diabetes diagnosis. The results of this study can help improve the efficiency of the methods of health professionals attempting to lower the rate of diabetes diagnoses.

## Background

According to the National Diabetes Statistics Report [NDSR] [1], 9.4% of the US total population have diabetes. The CDC also indicates that 33.9% of the US adult population is estimated to be pre-diabetes, with 90% unaware of their condition [2]. National surveillance data suggests that an estimated 11 out of 1000 Americans ages 45–64 will develop diabetes at nearly triple the incidence rate of the disease in individuals ages 18–44 [1]. The high proportions of diabetes are not only alarming but also conjure up important questions for practice efforts toward prevention and disease management, about the measurable connection between physical characteristics of the human body and the age at which the symptoms may arise or the age at diabetes diagnosis. These dimensional measurements are known as anthropometric measurements and are operationally defined by the National Health and Nutrition Examination Survey III [NHANES] as the “study of the measurement of the human body in terms of the dimensions of bone, muscle, and adipose tissue.” [3]

Body measurements are thought to be sound predictors for the development of diabetes in one’s lifetime [4]. Particularly, one measurable characteristic that is common in obesity studies is the use of the body mass index (BMI), which is used to “screen for weight categories that may lead to health problems such as diabetes.” [5] A study [6] noted that abdominal obesity, “is a major driver in the development of diabetes and cardiovascular disease.” The body measurement from the umbilicus to the back is considered the average sagittal abdominal diameter (SAD) [7]. The same study reported that among men, SAD had the strongest correlation of all of the studied variables, including scaling with the largest variation in insulin sensitivity, suggesting the strongest anthropometric association of any study.

Another measurement that is reported in studies focusing on the interaction between diabetes and anthropometric factors is waist circumference [8, 9]. According to a study [10], the waist circumference measurement is a statistically significant predictor of diabetes. Additionally, a previous research [11] found a positive correlation between waist circumference and diabetes risk using biomarkers typically connected to type-2 diabetes (T2D) diagnosis. Moreover, the upper leg length (ULL) has been found to be a key component in height contributing to the risk of diabetes [12]. While the current study will not focus on height, we will use ULL as an anthropometric factor to evaluate its association with age of diabetes diagnosis.

Diabetes is characterized as a disease of the metabolism and can create a hyperglycemic environment in the blood [13]. The American Diabetes Association (ADA) recommends that each person’s blood glucose target should be individualized based on certain existing health and lifestyle conditions [14]. The interaction between age and serum glucose levels in diabetes diagnosis is of important concern in the search to determine if there is a synergetic relationship between these variables. The literature suggests significant associations between anthropometric characteristics and diabetic complications. However, there still remains a major gap in the knowledge about the relative strengths of associations between specific anthropometric measures and age at diagnosis of diabetes. Our study elucidates the association between select anthropometric measurements, demographic variables, and age at diabetes diagnosis.

## Methods

### Data and subjects

**Fig. 1:**
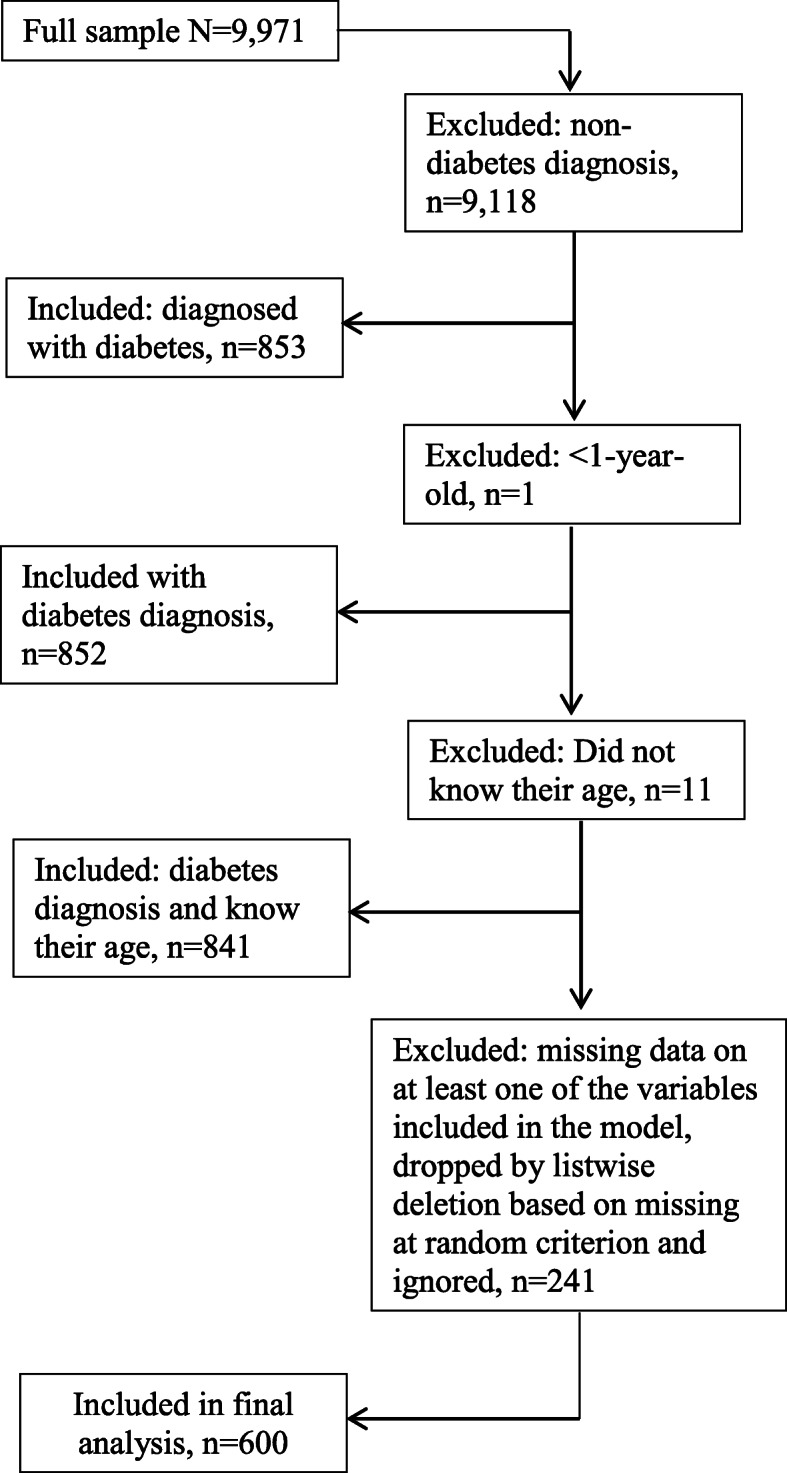
Inclusion/exclusion consort diagram

The present study uses the NHANES 2015–2016 data from the US Centers for Disease Control and Prevention [15]. The NHANES is a cross-sectional survey that monitors the health and nutritional status of the civilian noninstitutionalized U.S. population. It is a public use data depository which consists of large data sets collected from interviews conducted in respondents’ homes and standardized physical examinations in mobile examination centers. It contains data sets which can be described as demographic, laboratory, questionnaire and physical examination, including height and weight. To create the data for the present study, we merged laboratory examination data set (BIOPRO_I) containing glucose, refrigerated serum (mg/dL) with diabetes data set (DIQ_I) containing age at diagnosis with diabetes, Mobile Examination Center (MEC) Examination data set (BMX_I) containing body mass index, upper leg length, average sagittal abdominal diameter, waist circumference, and interview questionnaire data set (DEMO_I) containing demographic information including country of birth, gender, level of education, marital status, race, income, and household size. All respondents included in this study were both interviewed and examined at the Mobile Examination Center at the same time, indicated by the variable known as “Interview and examination status of the participant” as contained in the NHANES 2015–2016 Data Documentation, Codebook, and Frequencies [16]. Detailed procedures and methods which were used for collection of anthropometric measurements can be found in the NHANES Anthropometry Procedures Manual [17]. The full sample had 9971 individuals, of whom 853 individuals had been diagnosed with diabetes. One individual was excluded because they were less than a year old, 11 individuals who did not know their age were also excluded. In addition, we assumed that missing data for the variables of interest were missing at random and ignored them as recommended by the NHANES Analytic Guidelines, 2011–2014 and 2015–2016 if less than 10% of the data are missing. Therefore, the final sample size for analysis was 600 individuals (Fig. 1).

### Survey weights

The NHANES sample is selected through a complex, multistage probability design. To obtain reliable estimates for subgroups, NHANES oversamples persons who are identified as Hispanic, non-Hispanic Black, non-Hispanic Asian, non-Hispanic White and other persons aged 80 years and older at or below 185% of the Department of Health and Human Services (HHS) poverty guidelines [18]. Due to varying sampling probabilities of selection for respondents arising from clustering, the use of sampling weights and sample design variables is recommended for all analyses. Failing to account for the sampling parameters may lead to biased estimates, which are likely to overstate significance levels. We used MEC survey weight to adjust our estimates because DIQ data were merged with the MEC examination data and laboratory data.

### Outcome variable

The outcome variable was age at diabetes diagnosis in years, which was measured as continuous by data type and was self-reported during the lab and anthropometric data collection.

### Predictor variables

Consistent with previous studies predictor variables were selected as they related to the outcome variable, including continuous variables (i.e. BMI in kg/m^2^, ULL in cm, average SAD in cm, waist circumference in cm, total number of people in the household, and refrigerated glucose serum in mg/dL) [4, 19]. Additional categorical predictor variables such as country of birth, annual household income, level of education, gender, marital status, and race were included in the analysis. Specifically, the country of birth variable was categorized as follows: being born in the US and being born outside of the US. The annual household income had a category of under $20,000 and $20,000 and over. Gender was categorized as male and female. Marital status had six categories that included married, widowed, divorced, separated, never married, and living with partner. Race was categorized in terms of Mexican American, other Hispanic, non-Hispanic White, non-Hispanic Black, and other race that included multiracial groups.

### Statistical analysis

Descriptive analysis was performed for all variables. Means, standard errors, 95% confidence intervals (CI) and proportions are reported for continuous and categorical variables. In addition, weighted linear regression analysis was performed (after normality tests using histograms) to assess relationships between anthropometric and demographic factors and the age of diabetes diagnosis. Estimated effect sizes, *p*-values, confidence intervals are reported. We conducted sensitivity analysis for our sample estimates and those generated using Stata’s list-wise deletion to assess the effect of our missing data assumption. Data analysis was performed using Stata 12 (StataCorp, College Station, TX, USA).

## Results

### Study population

Table 1 summarizes weighted descriptive statistics for the study sample population (*n* = 600). The mean age for female participants was 1 year lower (47.8 years; 95% CI: 45.7–50.0 years) than male (48.8 years; 95% CI: 46.9–50.7 years). Fifty-seven percent of respondents were male, 57% were married, and 60% reported being non-Hispanic White. The means for different anthropometric indexes were as follows: Mean BMI was higher among female participants (33.8 kg/m^2^; 95% CI: 32.9–34.7 kg/m^2^) than male (31.5 kg/m^2^; 95% CI: 30.4–32.5 kg/m^2^), upper leg length was shorter among female participants (35.1 cm; 95% CI: 34.2–35.9 cm) than male (40.3 cm; 95% CI: 39.7–41.0 cm), average sagittal abdominal diameter was larger among female participants (26.0 cm; 95% CI: 25.3–26.6 cm) than male (25.7 cm; 95% CI: 24.9–26.6 cm), waist circumference was larger among females (111.4 cm; 95% CI: 109.6–113.2 cm) than males (110.9 cm; 95% CI: 108.1–113.8 cm), glucose serum was lower among females (139.8 mg/dL; 95% CI: 130.4–149.3 mg/dL) than males (153.7 mg/dL; 95% CI: 143.2–164.2 mg/dL). Among the respondents, 84% (*n* = 405) were born in the US and 16% (*n* = 195) outside of the US. The average household size, measured by the total number of people in the household, was three.

**Table 1:** Weighted characteristics and distribution of anthropometric measures in study sample (*n* = 600)

Continuous variables	Mean (Standard errors)			
	Male	Female	Difference between male and female	*p*-value
Age when first told you had diabetes (years) mean(se)	48.8 (0.9)	47.8 (1.0)	1.0	0.47
**Body Mass Index (kg/m**^**2**^)** mean(se)	**31.5 (0.5)**	**33.8 (0.4)**	**−2.3**	**0.00**
Upper leg length (cm)** mean(se)	**40.3 (0.3)**	**35.1 (0.4)**	**5.3**	**0.00**
Waist Circumference (cm) mean(se)	110.9 (1.3)	111.4 (0.8)	−0.4	0.76
Average Sagittal Abdominal Diameter (cm) mean (se)	25.7 (0.4)	26.0 (0.3)	−0.2	0.55
Glucose, refrigerated serum (mg/dL)* mean(se)	**153.7 (4.9)**	**139.8 (4.4)**	**13.9**	**0.05**
Total number of people in the Household	2.7			
Categorical variables	Categories (indicator)	*n*	Proportion	
Country of birth				
	Born in the US	405	0.84	
	Born outside of the US	195	0.16	
Annual household income				
	$ 0 to $ 19,999	148	0.15	
	$ 20,000 or more	452	0.85	
Gender				
	Male	336	0.57	
	Female	264	0.43	
Marital status				
	Married	353	0.57	
	Widowed	58	0.09	
	Divorced	73	0.11	
	Separated	20	0.03	
	Never married	57	0.12	
	Living with partner	39	0.08	
Race/Hispanic origin				
	Mexican American	137	0.11	
	Other Hispanic	90	0.06	
	Non-Hispanic White	165	0.60	
	Non-Hispanic Black	141	0.14	
	Other Race - Including Multi-Racial	67	0.09	
Education				
	Less than 9th grade	97	0.08	
	9-11th grade and 12th grade no diploma	78	0.11	
	High school graduate/GED or equivalent	137	0.21	
	Some college or AA degree	171	0.35	
	College graduate or above	117	0.25	

**p < 0.05

*p < 0.01

**Fig. 2:**
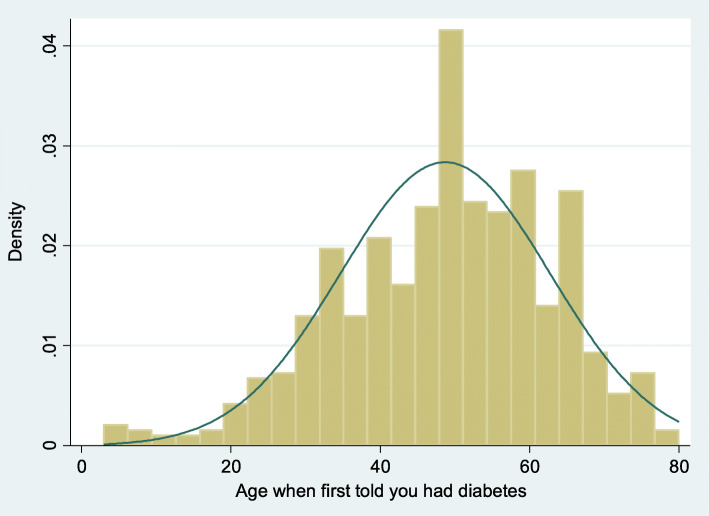
Histogram for the test of normality of age at diagnosis of diabetes

The weighted linear regression estimates are summarized in Table 2. The histogram for normality test showed that age at diagnosis of diabetes were approximately normally distributed (Fig. 2).

**Table 2:** Weighted linear regression estimates of predictor variables on outcome variable (age at diagnosis of diabetes)

			95% Confidence interval	
Age when first told you had diabetes	Coefficient	*p*-value	Lower limit	Upper limit
Body Mass Index*	**− 1.03**	**0.00**	**−1.40**	**−0.66**
Upper leg length (cm)*	**−0.91**	**0.00**	**−1.28**	**−0.54**
Average Sagittal Abdominal Diameter (cm)**	**1.24**	**0.02**	**0.20**	**2.27**
Waist Circumference (cm)	0.06	0.70	−0.25	0.36
Country of birth (born in the US)	−0.30	0.84	−3.44	2.84
Gender (Male)**	**3.41**	**0.03**	**0.36**	**6.45**
Education level - Adults 20+				
College graduate or above (referent)				
Less than 9th grade	−2.14	0.28	−6.21	1.92
9-11th grade and 12th grade no diploma	−2.54	0.29	−7.44	2.36
High school graduate/GED or equivalent**	**−5.40**	**0.01**	**−8.98**	**−1.81**
Some college or AA degree*	**−7.58**	**0.00**	**−11.33**	**−3.83**
Marital status				
Married (referent)				
Widowed	3.80	0.06	−0.15	7.74
Divorced	2.15	0.28	−1.92	6.23
Separated	−0.22	0.96	−9.54	9.99
Never married*	**−9.13**	**0.00**	**−14.86**	**− 3.40**
Living with partner	−2.53	0.20	−6.60	1.53
Race/Hispanic origin				
Non-Hispanic White (referent)				
Mexican American**	**− 5.00**	**0.02**	**−8.89**	**−1.12**
Other Hispanic	−3.02	0.18	−7.55	1.51
Non-Hispanic Black	−2.70	0.06	−5.54	0.13
Other Race - Including Multi-Racial	−0.74	0.77	−5.54	4.45
Annual household income				
$ 0 to $ 19,999 (referent)				
$ 20,000 or more	1.81	0.25	−5.92	5.05
Total number of people in the Household*	**−1.72**	**0.00**	**−2.42**	**− 1.02**
Glucose, refrigerated serum (mg/dL)**	**−0.02**	**0.01**	**− 0.03**	**− 0.01**
Constant	88.71	0.00	66.80	110.63

**p < 0.05

*p < 0.01

**Fig. 3:**
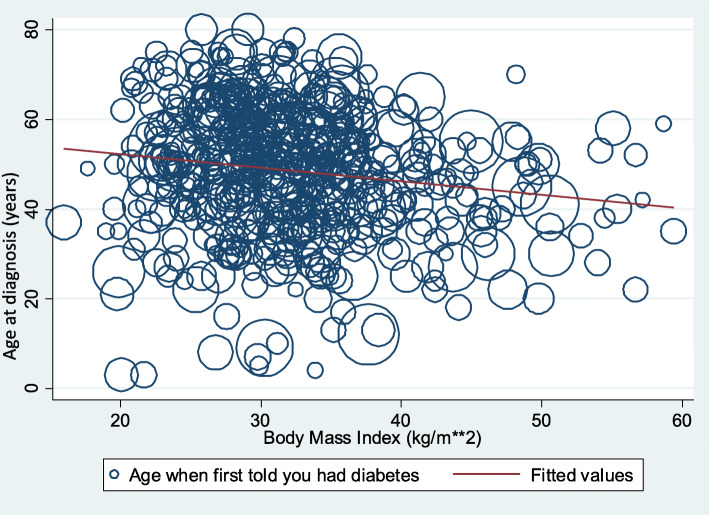
Weighted scatter plot of age at diagnosis and BMI

Specifically, the following anthropometric results yielded statistically significant results (*p* < 0.05). Age at diagnosis of diabetes is predicted to decrease by 1.03 years and 0.91 years when BMI and upper leg length go up by one unit each, respectively. In addition, a unit mg/dL increase in serum glucose was associated with a 0.02 years reduction in age of diagnosis with diabetes (*p <* 0.05). Conversely, age at diagnosis of diabetes increases by 1.24 years (later in life) when the average sagittal abdominal diameter goes up by 1 centimeter (cm) in our study population. For demographic predictors, age at diagnosis of diabetes for respondents whose level of education attainment was high school graduate/GED or equivalent and those who had some college or two-year college degrees reduced by 5.40 years and 7.58 years respectively compared to their counterparts who had college degree or higher level of degree (*p* < 0.05). In addition, male respondents were diagnosed with diabetes 3.41 years later than their female counterparts (*p* < 0.05). Respondents who had never married were diagnosed 9.13 years earlier than their married counterparts (*p <* 0.01*)*. Similarly, an increase in the size of a household by one person reduced age at diagnosis of diabetes by 1.72 years (*p* < 0.01). Mexican American respondents were diagnosed 5.00 (*p <* 0.05) years earlier than their non-Hispanic White counterparts. However, age at diagnosis of diabetes did not significantly change with the waist circumference (0.06 years, *p =* 0.70). Finally, age at diagnosis was inversely associated with BMI, as BMI increased, age at diagnosis decreased (Fig. 3).

Results of the sensitivity analysis showed consistent conclusions from our sample and those from list-wise deletion methodology implemented by Stata.

## Discussion

In this study, findings indicated that anthropometric measures are associated with age of diabetes diagnosis. In particular, as BMI increased by one unit, the age of diagnosis of diabetes decreased by 1.03. A previous study [20] found similar results when analyzing the difference in metabolic profiles between persons diagnosed with early type 2 diabetes (T2D) (age 18–44) compared to usual T2D diagnosis (≥ 45 years of age); their study showed an inverse linear relationship between BMI and age at diagnosis of T2D. These findings offer an insight for public health programs to intervene in younger age groups to avert the disease by targeting known major risk factors that precipitate development of T2D.

The upper leg length of the population was also a predictor variable for age of diabetes diagnosis. The relationship was statistically significant and showed that the age of diagnosis of diabetes decreased by 0.91 as the value of ULL (cm) increased by 1 unit. To our knowledge, this is one of the first studies to highlight the relationship between age of diabetes diagnosis and ULL, as most studies explored the association between leg length and the prevalence [21] or the incidence [22] (i.e., investigation did not focus on the age of diabetes diagnosis, as the outcome variable) of diabetes. Although our finding is noteworthy, it brings about questions of the correlations that ULL may provide for age of diabetes diagnosis among different study populations. When comparing the average ULL by gender, females had statistically significantly shorter upper leg length than males. Undoubtedly, knowing demographic or anthropometric predictors for the age of diabetes diagnosis is important, as it may inform clinical practice, intervention or policy.

Another statistically significant anthropometric predictor variable from our study is the average sagittal abdominal diameter, or length from the umbilicus to the back. Our study found that for every increase of 1 centimeter in the value of SAD, the age of diabetes diagnosis increased by 1.24 years. Related to the physiological symptoms of diabetes, other studies focusing on the relationship between SAD and insulin action have shown that SAD measurements had strong correlations with metabolic factors including insulin sensitivity and insulin resistance [7]. In addition to SAD, glucose serum is another sound anthropometric condition related to diabetes. Our findings also showed that albeit a small effect size, glucose serum was associated with a reduction in age at diagnosis with diabetes. In our study, waist circumference—a well-known diabetes predictor [10]—was surprisingly not statistically significant, perhaps due to the presence of specific characteristics in the population or there were confounders that we failed to control for in the analysis. Nevertheless, this particular discrepant finding suggests that further research is needed to shed light on the interaction between age of diabetes diagnosis measured continuously and waist circumference, since the latter is a screening indicator for diabetes [23].

Other predictor variables that were evaluated in our study were socio-demographic factors (gender, race, occupation, place of birth, etc.). In our analysis, age at diagnosis of diabetes is predicted to be lower for the following individuals: never married, high school graduates or equivalent or those with some college or 2-year college graduates and Mexican Americans. Efforts should be made to ensure that individuals at risk have access to diabetes prevention strategies to mitigate the probability of developing the disease. Concerning gender, age at diagnosis of diabetes is predicted to be higher in male respondents than in female, plausibly due to gestational diabetes characterizing certain women.

Despite the soundness of the current study, it has limitations that we would like to highlight. A major limitation of this study is that it uses cross-sectional survey data. Cross-sectional designs can result in biases such as non-response bias and recall bias. In this study, recall bias could be a factor based on individuals who were interviewed not remembering the age they were first told they had diabetes. Nevertheless, one of the strengths of this study is that it used a nationally representative population–based survey to assess the associations in the variables of interest.

## Conclusion

In conclusion, this study’s findings suggest that anthropometric indexes are salient indicators related to age at diagnosis of diabetes among the population studied. Using a nationally representative dataset, our study contributes to filling the gap in collective knowledge regarding the effect of common anthropometric measurements and age at diagnosis of diabetes. The public health significance of determining the relative associations between these factors and age at diagnosis of diabetes cannot be overstated, given the steady rise in the proportion of diagnoses in the US and globally. These findings can be used in clinical and epidemiological settings to improve the efficiency of the targeted efforts by public health professionals fighting against the rising trend of diabetes diagnoses. Furthermore, the results of this weighted linear regression analysis between these predictor variables and outcome variable show the relative weight that each anthropometric and demographic measure has regarding the age of diabetes diagnosis. It gives public health and allied health professionals opportunities to aggregate their tasks for the most effective outcomes.

## Data Availability

The datasets used and/or analyzed during the current study are available in the CDC NHANES repository, https://www.cdc.gov/nchs/nhanes/Index.htm

## Abbreviations

NHANES: National Health and Nutrition Examination Survey
BMI: Body Mass Index
NDSR: National Diabetes Statistics Report
CDC: Centers for Disease Control and Prevention
SAD: Sagittal Abdominal Diameter
T2D: Type-2 Diabetes
ULL: Upper Leg Length
ADA: American DiabetesAssociation
BIOPRO_I: Laboratory examination data set
DIQ_I: Diabetes data set
MEC: Mobile Examination Center
BMX_I: Examination data set
DEMO_I: Interview questionnaire data set
HHS: Department of Health and Human Services
CI: Confidence Intervals
